# Development and validation of a machine learning model for survival risk stratification after esophageal cancer surgery

**DOI:** 10.3389/fonc.2022.1068198

**Published:** 2022-12-09

**Authors:** Jinye Xu, Jianghui Zhou, Junxi Hu, Qinglin Ren, Xiaolin Wang, Yusheng Shu

**Affiliations:** ^1^ Clinical Medical College, Yangzhou University, Yangzhou, China; ^2^ Department of Thoracic Surgery, Northern Jiangsu People’s Hospital Affiliated to Yangzhou University, Yangzhou, China

**Keywords:** esophageal neoplasms, machine learning, surgery, prognosis, survival risk stratification

## Abstract

**Background:**

Prediction of prognosis for patients with esophageal cancer(EC) is beneficial for their postoperative clinical decision-making. This study’s goal was to create a dependable machine learning (ML) model for predicting the prognosis of patients with EC after surgery.

**Methods:**

The files of patients with esophageal squamous cell carcinoma (ESCC) of the thoracic segment from China who received radical surgery for EC were analyzed. The data were separated into training and test sets, and prognostic risk variables were identified in the training set using univariate and multifactor COX regression. Based on the screened features, training and validation of five ML models were carried out through nested cross-validation (nCV). The performance of each model was evaluated using Area under the curve (AUC), accuracy(ACC), and F1-Score, and the optimum model was chosen as the final model for risk stratification and survival analysis in order to build a valid model for predicting the prognosis of patients with EC after surgery.

**Results:**

This study enrolled 810 patients with thoracic ESCC. 6 variables were ultimately included for modeling. Five ML models were trained and validated. The XGBoost model was selected as the optimum for final modeling. The XGBoost model was trained, optimized, and tested (AUC = 0.855; 95% CI, 0.808-0.902). Patients were separated into three risk groups. Statistically significant differences (p < 0.001) were found among all three groups for both the training and test sets.

**Conclusions:**

A ML model that was highly practical and reliable for predicting the prognosis of patients with EC after surgery was established, and an application to facilitate clinical utility was developed.

## Introduction

Among all malignant tumors, the incidence and mortality rates for EC rank seventh and sixth, respectively, worldwide. In terms of pathological type, the most common type of EC is ESCC (1). ESCC dominates in China, which accounts for half of the world’s cases of ESCC (2). The prognosis for patients with EC varies considerably due to different clinicopathological stages and treatment options (3). For patients with resectable EC, surgical resection is the preferred therapy option (4). The patient information gathered during the procedure can be used to more accurately evaluate the patient’s prognosis. Effective prediction of patient prognosis following radical esophagectomy can provide an important reference for patient’s postoperative treatment plans, supporting individualized management of EC.

As medical data proliferates and technology and artificial intelligence develop at a rapid pace, using big data analysis to construct survival prediction models has become an important research topic. ML is a sub-field of artificial intelligence, which is the practice of developing systems that learn from data to identify categories and provide precise predictions of future events (5). In medicine, it can be deployed in clinical databases to develop valid risk models and redefine patient categories (6). ML approaches have been utilized in the construction of prognostic models for a variety of malignancies such as lung, breast, liver, and gastrointestinal cancers (7–10), showing great predictive efficacy and demonstrating important clinical value.

TNM staging is the most extensively used approach for assessing the prognosis of patients with EC, although it is challenging to produce individualized and accurate prediction since it excludes other prognostic-related features (11). As a result, in this work, we used COX regression to screen prognostic risk factors and created an ML model based on them to stratify survival risk in patients with EC following surgery. It was hoped that this would provide a new approach to formulating a postoperative treatment plan and assessing the prognosis of these patients.

## Methods

### Data collection

The clinicopathological characteristics and follow-up data of patients with thoracic ESCC who received radical esophagectomy at the Department of Thoracic Surgery, Northern Jiangsu People’s Hospital from January 2014 to June 2017 were analyzed. The overall survival (OS), defined as the time interval from the date of surgery to the end of the study or patient death, was the major predictive outcome for this study. Patients were followed up by telephone or outpatient after they were discharged from the hospital. They had a follow-up visit every 3 months for the first 2 years after surgery, once every 6 months for 2-5 years, and once a year after 5 years. The follow-up was carried out until June 2022. All patients were staged by pathology (pTNM) after surgery. A total of 16 clinicopathological characteristics were included. They included gender, age, type of surgery, hypertension, diabetes, smoking, drinking, tumor size, tumor center location, histological grade, pT stage, pN stage, vascular invasion, nerve violations, pathological type, and surgical margin. All clinicopathological characteristics were easily obtained from the patient’s records.

The inclusion criteria were: (1) no antitumor therapy before surgery such as chemoradiotherapy, immunotherapy, and targeted therapy, (2) liver, lung, brain, and other distant metastases were excluded by preoperative CT, magnetic resonance imaging, bone scanning, color ultrasound, or other examinations before surgery, (3) the anatomical center of the tumor was located in the thoracic segment, (4) patients underwent transthoracic radical esophagectomy and were diagnosed with ESCC by postoperative pathological examination, and (5) the clinicopathological files and follow-up data were complete. Exclusion criteria were: (1) other pathological tissue types on postoperative pathology, (2) a history of other malignant tumors before surgery, (3) postoperative survival of less than 30 days, and (4) lost to follow-up.

### Model training and testing

COX regression was used in the training set to screen the variables impacting OS, and the hazard ratio (HR) and 95% confidence interval (CI) were determined. Based on the final features incorporated into the modeling, 5 ML models including Decision Tree, random forest (RF), support vector machine (SVM), gradient boosting machine (GBM), and XGBoost were trained and validated using 4 × 5-fold nCV(four outer iterations and five inner iterations). nCV provides a more accurate estimate of the validation error of a model on unknown datasets by averaging its performance metrics (12). Based on the average performance of each ML model, the optimum model is chosen for the final modeling.

All patients were randomly divided into a training set (567 patients) and a test set (243 patients) by computer in a 7:3 ratio. Based on the data of the training set, the grid search method was used to search the parameters, and the model was trained and verified internally by combining the 5-fold CV, thus completing the optimization of the model. Subsequently, the model was used for survival risk stratification and survival analysis of patients with EC and validated with an independent test set. A receiver operating characteristic (ROC) curve was plotted, and AUC, accuracy (ACC), and F1-Score (meaning the weighted average of precision and recall (13)) were calculated to evaluate the model performance. A calibration curve was plotted to evaluate the fitting of the model. Decision curve analysis (DCA), which calculates the net benefit of each strategy at each level of threshold probability (14) relating to the application of the model, was used to determine clinical utility (15). The X-tile software was used to confirm the optimal cut-off point for the survival probability values predicted by the model in the training set, and the patients were divided into different risk groups, and the test set was categorized using the same grouping criteria. We used the Kaplan-Meier methods and the log-rank test to perform survival analysis on the training set, and we verified it on the test set.

### Statistical analysis

The data were analyzed using R software (version 4.1.3) and SPSS software (version 25.0).

The training, testing and optimization of ML models are mainly implemented through the mlr package. The ROC curve, calibration curve, DCA, and survival curve are implemented by the pROC package, the Predtools package, the Dcurves package, and the Survminer package, respectively. The online calculator is implemented through the Shiny package. The mean (SD, standard deviation) was employed to describe normally distributed measurement data, and the independent samples *t* test was utilized to compare groups. The categorical data were described by frequency (percentage), the *Mann-Whitney U* test was used to compare the ordered categorical data between groups, and the *χ2* test was used to analyze the unordered categorical data between groups. The optimal cut-off point for survival risk stratification was achieved by X-Tile software (version 3.6.1). A p-value ≤ 0.05 was specified as statistically significant.

## Results

### Basic information

A total of 810 patients with ESCC were enrolled. There were 611 (75.4%) males and 199 (24.5%) females. The age distribution was 63.4 ± 6.97 (range: 41–84) years, and the median follow-up time was 66 months (range: 1-83 months). The 5-year postoperative OS rate for the training set was 66.6%, and the 5-year postoperative OS rate for the test set was 64.1%. The clinicopathological characteristics of the training and test sets were compared and all *p*-values were >0.05 (**Table 1**).

**Table 1:** Comparison of clinicopathological characteristics between the training and test sets.

	Training (N=567)	Test (N=243)	P-value
**Gender**			
Male	429 (75.7%)	182 (74.9%)	0.887
Female	138 (24.3%)	61 (25.1%)	
**Age (years)**			
Mean (SD)	63.4 (6.94)	63.5 (7.04)	0.751
Median [Min, Max]	64.0 [41.0, 79.0]	64.0 [45.0, 84.0]	
**Type of surgery**			
Sweet Esophagectomy	28 (4.9%)	15 (6.2%)	0.344
Ivor-Lewis Esophagectomy	16 (2.8%)	9 (3.7%)	
Thoracoscopic Esophagectomy	154 (27.2%)	77 (31.7%)	
McKeown Esophagectomy	369 (65.1%)	142 (58.4%)	
**Hypertension**			
Yes	174 (30.7%)	71 (29.2%)	0.738
No	393 (69.3%)	172 (70.8%)	
**Diabetes**			
Yes	74 (13.1%)	22 (9.1%)	0.135
No	493 (86.9%)	221 (90.9%)	
**Smoking**			
Yes	255 (45.0%)	108 (44.4%)	0.951
No	312 (55.0%)	135 (55.6%)	
**Drinking**			
Yes	209 (36.9%)	94 (38.7%)	0.68
No	358 (63.1%)	149 (61.3%)	
**Tumor size (cm)**			
≤4	336 (59.3%)	148 (60.9%)	0.851
4-8	208 (36.7%)	80 (32.9%)	
≥8	23 (4.1%)	15 (6.2%)	
**Tumor Centre Location**			
Upper thoracic segment	30 (5.3%)	10 (4.1%)	0.77
Middle thoracic segment	222 (39.2%)	95 (39.1%)	
Lower thoracic segment	315 (55.6%)	138 (56.8%)	
**Histological grade**			
G1	59 (10.4%)	19 (7.8%)	0.239
G2	389 (68.6%)	167 (68.7%)	
G3	119 (21.0%)	57 (23.5%)	
**PT stage**			
T1	178 (31.4%)	72 (29.6%)	0.510
T2	129 (22.8%)	52 (21.4%)	
T3	141 (24.9%)	66 (27.2%)	
T4	119 (21.0%)	53 (21.8%)	
**pN stage**			
N0	383 (67.5%)	152 (62.6%)	0.219
N1	117 (20.6%)	61 (25.1%)	
N2	55 (9.7%)	25 (10.3%)	
N3	12 (2.1%)	5 (2.1%)	
**Vascular invasion**			
Yes	70 (12.3%)	35 (14.4%)	0.493
No	497 (87.7%)	208 (85.6%)	
**Nerve violations**			
Yes	69 (12.2%)	37 (15.2%)	0.285
No	498 (87.8%)	206 (84.8%)	
**Pathological types**			
Superficial type	175 (30.9%)	71 (29.2%)	0.279
Medullary type	226 (39.9%)	114 (46.9%)	
Fungating type	12 (2.1%)	6 (2.5%)	
Ulcerative type	149 (26.3%)	49 (20.2%)	
Infiltrating type	5 (0.9%)	3 (1.2%)	
**Surgical margin**			
Yes	19 (3.4%)	6 (2.5%)	0.658
No	548 (96.6%)	237 (97.5%)	
**Survival status**			
Dead	190 (33.5%)	87 (35.8%)	0.583
Alive	377 (66.5%)	156 (64.2%)	

### Univariate and multivariate analysis

In this study, variable screening was performed by COX regression analysis. In univariate regression, age, type of surgery, hypertension, smoking, tumor size, histological grade, pT stage, pN stage, vascular invasion, nerve violations, and pathological types were all associated with OS (P-value < 0.05). Factors with statistically significant differences in the univariate analysis were integrated into the multifactorial regression and forward selection was performed, which showed that age, hypertension, smoking, histological grade, pT stage, and pN stage were independent predictors of OS (P-value < 0.05, **Table 2**).

**Table 2:** Risk factor analysis of patients with EC in the training set.

Variable	Univariate analyses		Multivariate analyses	
	HR (95%CI)	p-value	HR (95%CI)	p-value
**Gender**		0.233		
male	0.823(0.597 - 1.134)			
female	Reference			
**Age (years)**		0.015		0.011
≤64	Reference		Reference	
65-69	0.868(0.606 - 1.242)		1.028(0.712- 1.486)	
≥70	1.555(1.101 - 2.197)		1.732(1.211 - 2.476)	
**Type of surgery**		0.021		0.560
Sweet Esophagectomy	1.947(1.139 - 3.330)			
Ivor-Lewis Esophagectomy	1.993(1.012 - 3.922)			
Thoracoscopic Esophagectomy	0.860(0.607 - 1.217)			
McKeown Esophagectomy	Reference			
**Hypertension**		<0.001		0.028
Yes	1.675(1.253 - 2)		1.403(1.038 - 1.897)	
No	Reference		Reference	
**Diabetes**		0.766		
Yes	1.064(0.708- 1.599)			
No	Reference			
**Smoking**		0.006		0.044
Yes	1.494(1.122 - 1.988)		1.356(1.008 - 1.823)	
No	Reference		Reference	
**Drinking**		0.198		
Yes	1.210(0.905- 1.619)			
No	Reference			
**Tumor size (cm)**		0.017		0.770
≤4	Reference			
4-8	1.517(1.132 - 2.034)			
≥8	1.552(0.784 - 3.073)			
**Tumor Centre Location**		0.107		
Upper thoracic segment	Reference			
Middle thoracic segment	1.575(0.683- 3.634)			
Lower thoracic segment	2.117(0.932- 4.810)			
**Histological grade**		<0.001		0.003
G1	Reference		Reference	
G2	1.855(1.000 - 3.441)		2.244(1.202 - 4.188)	
G3	3.608(1.894 - 6.870)		2.754(1.438 - 5.276)	
**pT stage**		<0.001		0.006
T1	Reference		Reference	
T2	1.971(1.206 - 3.221)		1.279(0.768- 2.129)	
T3	3.890(2.501 - 6.051)		2.090(1.294- 3.374)	
T4	3.954(2.514 - 6.220)		1.922(1.176 - 3.142)	
**pN stage**		<0.001		<0.001
N0	Reference		Reference	
N1	3.715(2.640 - 5.226)		3.025(2.089 - 4.380)	
N2	8.249(5.643 - 12.059)		5.342(3.469 - 8.226)	
N3	12.995(6.839 - 24.695)		9.771(4.937 - 19.338)	
**Vascular invasion**		<0.001		0.443
Yes	2.688(1.905 - 3.792)			
No	Reference			
**Nerve violations**		0.010		0.563
Yes	1.652(1.129 - 2.417)			
No	Reference			
**Pathological types**		<0.001		0.222
Superficial type	Reference			
Medullary type	3.355(2.171 - 5.184)			
Fungating type	2.840(0.991 - 8.139)			
Ulcerative type	3.471(2.197 - 5.483)			
Infiltrating type	5.885(1.780 - 19.461)			
**Surgical margin**		0.659		
Yes	1.185(0.557- 2.522)			
No	Reference			

### Model training and testing

Ultimately, 6 variables including age, hypertension, smoking, histological grade, pT stage, and pN stage were selected for modeling. Based on these 6 variables, each ML model was trained and verified by 4 × 5-fold nCV, and the average performance is noted in **Table 3**. The average AUC of each model in external validation was: Decision Tree (AUC = 0.763), SVM (AUC = 0.809), RF (AUC = 0.809), GBM (AUC = 0.830), and XGBoost (AUC = 0.831). After a comprehensive comparison of several performance indicators, XGBoost was chosen as the final model.

**Table 3:** Comparison of the models’ average performance in nested cross-validation.

	AUC		ACC		F1-Score	
	Training	Test	Training	Test	Training	Test
Decision tree	0.814	0.763	0.799	0.758	0.856	0.826
SVM	0.848	0.809	0.781	0.758	0.848	0.832
RF	0.862	0.809	0.787	0.741	0.858	0.829
GBM	0.844	0.830	0.785	0.766	0.847	0.831
XGBoost	0.854	0.831	0.795	0.770	0.854	0.835

**Figure 1:**
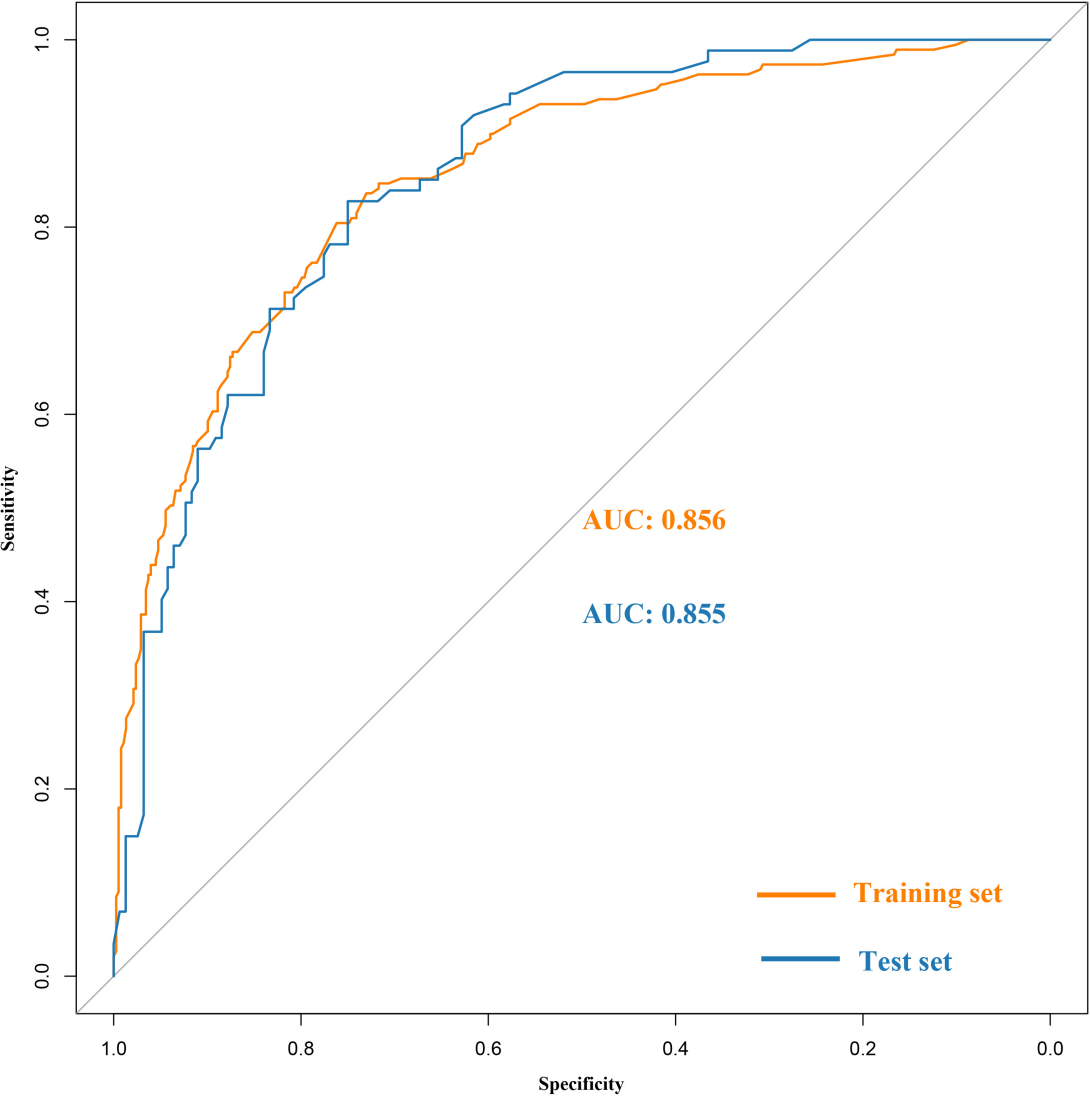
Receiver operating characteristic (ROC) curves of the XGBoost model in the training and the test set.

**Figure 2:**
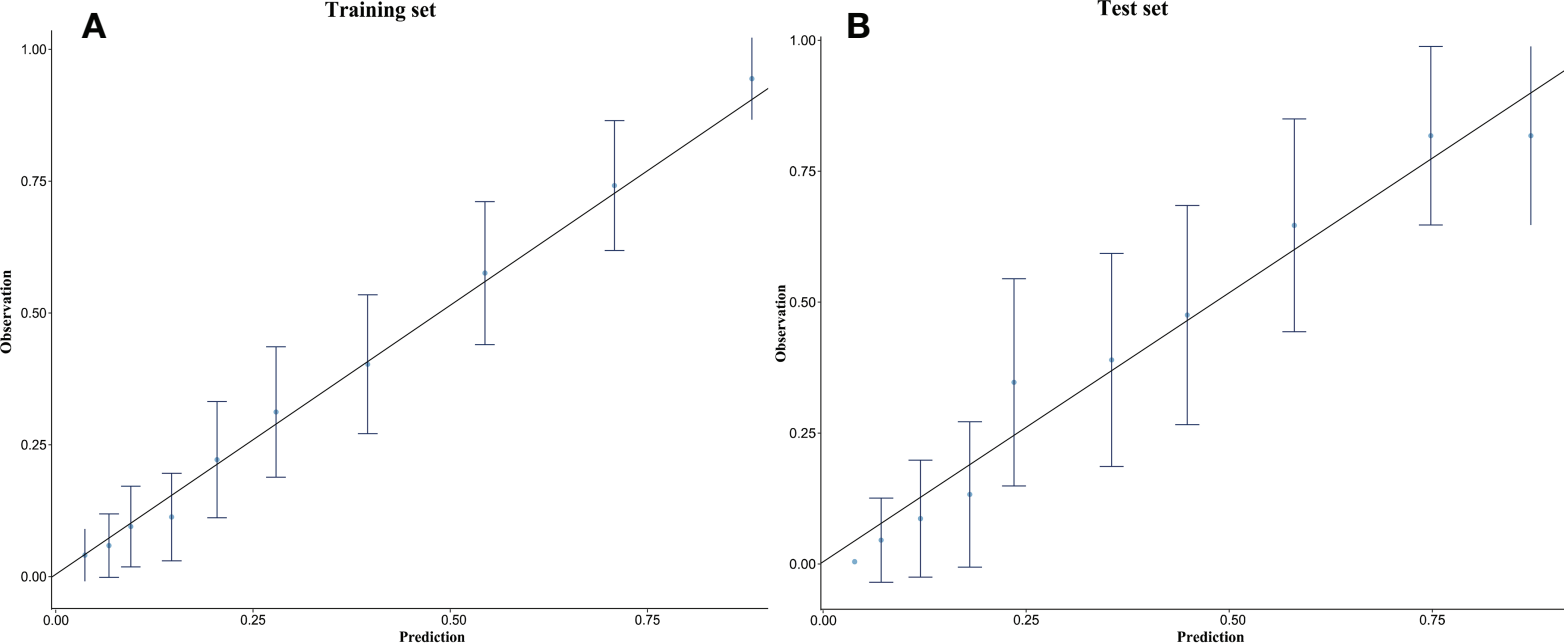
Calibration curves of the XGBoost model in the training set **(A)** and the test set **(B)**.

**Figure 3:**
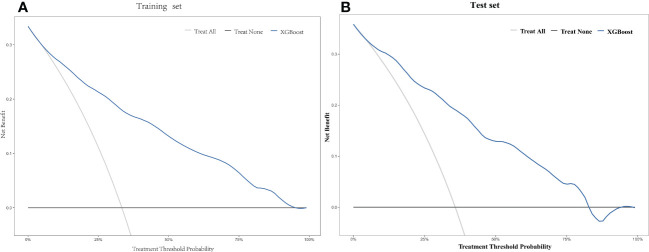
Decision curve analysis (DCA) of the XGBoost model in the training set **(A)** and the test set **(B)**.

Based on the training set, the model was optimized. The XGBoost model was validated on the test cohort, and the ROC curve was plotted (**Figure 1**). The model’s performance on the test cohort was calculated (AUC = 0.855; 95% CI, 0.808-0.902). The calibration curves (**Figure 2**) were plotted, which demonstrated that the predicted results of the model were consistent with the observed results, both in the training and test sets. DCA (**Figure 3**) was plotted, the area between the XGBoost model curve and the “Treat None” or “Treat All” line represents the clinical utility of the model, the farther the model curve is from the “Treat None” or “Treat All” line, the better the clinical value. In both training and test sets, DCA indicated that the clinical utility of the model was high.

### Risk stratification

**Figure 4:**
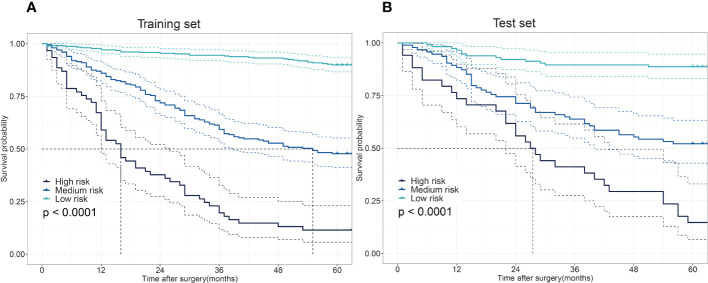
The differences in the overall survival (OS) among low-, medium-, and high-risk patients. Survival disparities among different risk groups in the training set **(A)** and the test set **(B)**.

The 5-year OS probability predicted by the XGBoost model in the training set was taken as the model score. Those with scores ≤0.21; 0.22–0.75; ≥0.76 were categorized as three different risk groups. Patients in the training cohort were divided into different risk groups: high-risk (61 patients), medium-risk (199 patients), and low-risk (307 patients). The 5-year OS rate for patients predicted to be high-risk by the XGBoost model was 11.4%, those predicted to be medium-risk was 47.7%, and those predicted to be low-risk was 89.9%. The difference was statistically significant (χ² = 190.284; p < 0.001). In the test set, patients were divided into risk groups with the same classification criteria as the training set: high-risk (34 patients), medium-risk (94 patients), and low-risk (115 patients). The 5-year OS rate for patients predicted to be high-risk by the model was 14.7%, for those predicted to be medium-risk was 52.1%, and for those predicted to be low-risk was 88.6%. The difference was statistically significant (χ² = 72.220; p < 0.001) (**Figure 4**).

### Development of applications

It is crucial that the constructed forecasting model can be simply applied in practice. A user-friendly application program (https://eso-predict.shinyapps.io/shiny_mlr/) was developed for both patients and clinicians. The application is offered as a web page with a backend that invokes the trained XGBoost model. The user enters the necessary information in response to the prompts and then clicks the “Predict” button to obtain the 5-year survival risk following EC surgery.

## Discussion

Accurate surgical prognosis prediction is critical for subsequent therapy decisions in patients with EC. At present, prognosis prediction following EC surgery is mainly based on COX regression modeling (11, 16), which assumes a linear association between outcomes and variables, and thus, it cannot capture the nonlinear relationship between various characteristics and outcomes (17). In contrast, ML techniques can better capture the complex associations between features and outcomes (18), thus improving the accuracy of the model. However, the predictive process of ML models is poorly interpretable, reducing the trust of patients and physicians in the models (19). This study screened the final modeled risk factors through COX univariate and multivariate analysis, which increased the interpretability to a certain extent. Previously (3), ML methods have been used to build a ML model to predict the prognosis of patients with EC based on information from the Surveillance, Epidemiology, and End Results(SEER) database. However, this is a public database, and whether models from a public database can be used locally needs further verification. Moreover, its prediction model incorporates a total of 24 features, some of which are difficult to obtain in clinical practice, and this reduces the practicality and reliability of the model in clinical practice.

This study observed 5-year OS after surgery by a long-term follow-up of patients who underwent radical EC surgery at a single institution. A total of 16 characteristics commonly found in the records of patients with EC that might have an impact on their prognosis were collected. Multifactorial analysis identified age, hypertension, smoking, histological grading, pT stage and pN stage as independent predictors of OS. ML models based on these 6 clinicopathological characteristics were developed to predict the 5-year survival status of patients following EC surgery. Among these 6 characteristics, age and smoking were common risk factors for EC (20). Studies have shown that patients with hypertension at the time of cancer diagnosis have a higher all-cause mortality rate than those without hypertension especially in patients with longer follow-up (21). Histological grade, pT stage, and pN stage are widely recognized to influence the prognosis of patients with EC (22–24). In this study, to prevent overfitting of ML models, meaning that they perform well in training but poorly in testing (25), each ML model was trained and validated by nCV to estimate its prediction performance more accurately. Synthesizing the performance of each ML model, XGBoost was ultimately selected as the best model for final modeling.

CV was applied to the training set for hyperparameter tuning, and risk stratification and survival analysis were performed on the training set. Risk stratification and survival analysis were performed on the test cohort using the same partitioning criteria as the training cohort. The results show significant differences in survival among the different risk groups in both the training and test cohorts. In this study, 54.1% of patients in the training set and 47.3% in the test set were classified as low-risk, and the 5-year OS rates of the low-risk group in the training and test cohorts were 89.9% and 88.6%, respectively. For this reason, patients in the low-risk group may not need adjuvant therapy following surgery. In contrast, patients in this study classified as being in the medium–high risk group should receive more aggressive adjuvant therapy following surgery. ML algorithms are intricate and cannot be applied clinically through scoring or nomogram plots. To facilitate the use of this model in clinical decision-making, an application was developed to provide rapid access to the predictive ability of the XGBoost model. Clinicians only need to input data on these 6 variables to obtain the 5-year survival probabilities and risk stratification predicted by this model, so that the XGBoost model constructed in this study is highly practical and reliable.

This study has several limitations. First, this was a single-center study with a limited number of patients, and ML models derived from a larger dataset could achieve more accurate results (26). Therefore, in subsequent studies, multi-center data could be added for training and external validation to obtain a more reliable prediction model. Furthermore, this study was developed and validated utilizing retrospective files, and prospective validation studies should be performed to confirm the reliability of this model before it enters formal clinical practice.

## Conclusions

In conclusion, this study constructed a ML model for predicting the risk of 5-year OS following EC surgery based on 6 common clinicopathological characteristics. The XGBoost model had the best performance of the several models tested. The XGBoost model can provide an important reference for prognostic assessment and postoperative treatment decisions for patients with EC thus promoting the individualized management of EC.

## Data availability statement

The raw data supporting the conclusions of this article will be made available by the authors, without undue reservation.

## Ethics statement

The studies involving human participants were reviewed and approved by Medical Ethics Committee of Northern Jiangsu People’s Hospital. Written informed consent for participation was not required for this study in accordance with the national legislation and the institutional requirements.

## Author contributions

JX was involved in the study design, statistical analysis, and writing the first draft of the paper; JZ, JH, and QR were responsible for data collection and collation; XW was responsible for the study design, proofreading and paper revision; and YS was responsible for the study design, manuscript review and revision. All authors have read and agreed to the published version of the manuscript.

## References

[1] Bray F Ferlay J Soerjomataram I Siegel RL Torre LA Jemal A. Global cancer statistics 2018: GLOBOCAN estimates of incidence and mortality worldwide for 36 cancers in 185 countries. CA: Cancer J Clin (2018) 68:394–424. doi: 10.3322/caac.21492 30207593

[2] Abnet CC Arnold M Wei WQ. Epidemiology of esophageal squamous cell carcinoma. Gastroenterology (2018) 154:360–73. doi: 10.1053/j.gastro.2017.08.023 PMC5836473 28823862

[3] Gong X Zheng B Xu G Chen H Chen C. Application of machine learning approaches to predict the 5-year survival status of patients with esophageal cancer. J Thorac Dis (2021) 13:6240–51. doi: 10.21037/jtd-21-1107 PMC8662490 34992804

[4] Waters JK Reznik SI. Update on management of squamous cell esophageal cancer. Curr Oncol Rep (2022) 24:375–85. doi: 10.1007/s11912-021-01153-4 35142974

[5] Verma AA Murray J Greiner R Cohen JP Shojania KG Ghassemi M. Implementing machine learning in medicine. CMAJ Can Med Assoc J = J l'Association medicale Can (2021) 193:E1351–E7. doi: 10.1503/cmaj.202434 PMC8432320 35213323

[6] Deo RC. Machine learning in medicine. Circulation (2015) 132:1920–30. doi: 10.1161/CIRCULATIONAHA.115.001593 PMC5831252 26572668

[7] Lynch CM Abdollahi B Fuqua JD de Carlo AR Bartholomai JA Balgemann RN. Prediction of lung cancer patient survival *via* supervised machine learning classification techniques. Int J Med Inf (2017) 108:1–8. doi: 10.1016/j.ijmedinf.2017.09.013 PMC5726571 29132615

[8] Zhou CM Xue Q Wang Y Tong J Ji M Yang JJ. Machine learning to predict the cancer-specific mortality of patients with primary non-metastatic invasive breast cancer. Surg Today (2021) 51:756–63. doi: 10.1007/s00595-020-02170-9 33104877

[9] Ji GW Fan Y Sun DW Wu MY Wang K Li XC. Machine learning to improve prognosis prediction of early hepatocellular carcinoma after surgical resection. J hepatocellular carcinoma (2021) 8:913–23. doi: 10.2147/JHC.S320172 PMC8370036 34414136

[10] Christopherson KM Das P Berlind C Lindsay WD Ahern C Smith BD. A machine learning model approach to risk-stratify patients with gastrointestinal cancer for hospitalization and mortality outcomes. Int J Radiat oncology biology Phys (2021) 111:135–42. doi: 10.1016/j.ijrobp.2021.04.019 33933480

[11] Liu X Guo W Shi X Ke Y Li Y Pan S. Construction and verification of prognostic nomogram for early-onset esophageal cancer. Bosn J Basic Med Sci (2021) 21:760–72. doi: 10.17305/bjbms.2021.5533 PMC8554706 33823125

[12] Maros ME Capper D Jones DTW Hovestadt V von Deimling A Pfister SM. Machine learning workflows to estimate class probabilities for precision cancer diagnostics on DNA methylation microarray data. Nat Protoc (2020) 15:479–512. doi: 10.1038/s41596-019-0251-6 31932775

[13] Munir K Elahi H Ayub A Frezza F Rizzi A. Cancer diagnosis using deep learning: A bibliographic review. Cancers (2019) 11(9):1235. doi: 10.3390/cancers11091235 31450799 PMC6770116

[14] Vickers AJ Holland F. Decision curve analysis to evaluate the clinical benefit of prediction models. Spine J Off J North Am Spine Soc (2021) 21:1643–8. doi: 10.1016/j.spinee.2021.02.024 PMC8413398 33676020

[15] Vickers AJ Elkin EB. Decision curve analysis: a novel method for evaluating prediction models. Med decision making an Int J Soc Med Decision Making (2006) 26:565–74. doi: 10.1177/0272989X06295361 PMC2577036 17099194

[16] Tang X Zhou X Li Y Tian X Wang Y Huang M. A novel nomogram and risk classification system predicting the cancer-specific survival of patients with initially diagnosed metastatic esophageal cancer: A SEER-based study. Ann Surg Oncol (2019) 26:321–8. doi: 10.1245/s10434-018-6929-0 30357578

[17] Kim H Park T Jang J Lee S. Comparison of survival prediction models for pancreatic cancer: Cox model versus machine learning models. Genomics Inform (2022) 20:e23. doi: 10.5808/gi.22036 35794703 PMC9299568

[18] Buch VH Ahmed I Maruthappu M. Artificial intelligence in medicine: current trends and future possibilities. Br J Gen Pract J R Coll Gen Practitioners (2018) 68:143–4. doi: 10.3399/bjgp18X695213 PMC5819974 29472224

[19] Moncada-Torres A van Maaren MC Hendriks MP Siesling S Geleijnse G. Explainable machine learning can outperform cox regression predictions and provide insights in breast cancer survival. Sci Rep (2021) 11:6968. doi: 10.1038/s41598-021-86327-7 33772109 PMC7998037

[20] Li S Chen H Man J Zhang T Yin X He Q. Changing trends in the disease burden of esophageal cancer in China from 1990 to 2017 and its predicted level in 25 years. Cancer Med (2021) 10:1889–99. doi: 10.1002/cam4.3775 PMC7940228 33586344

[21] Petrelli F Ghidini A Cabiddu M Perego G Lonati V Ghidini M. Effects of hypertension on cancer survival: A meta-analysis. Eur J Clin Invest (2021) 51:e13493. doi: 10.1111/eci.13493 33470426

[22] Shahbaz Sarwar CM Luketich JD Landreneau RJ Abbas G. Esophageal cancer: an update. Int J Surg (2010) 8:417–22. doi: 10.1016/j.ijsu.2010.06.011 20601255

[23] Yang J Lu Z Li L Li Y Tan Y Zhang D. Relationship of lymphovascular invasion with lymph node metastasis and prognosis in superficial esophageal carcinoma: systematic review and meta-analysis. BMC Cancer (2020) 20:176. doi: 10.1186/s12885-020-6656-3 32131772 PMC7057611

[24] Gupta V Coburn N Kidane B Hess KR Compton C Ringash J. Survival prediction tools for esophageal and gastroesophageal junction cancer: A systematic review. J Thorac Cardiovasc Surg (2018) 156:847–56. doi: 10.1016/j.jtcvs.2018.03.146 30011772

[25] Ji GW Jiao CY Xu ZG Li XC Wang K Wang XH. Development and validation of a gradient boosting machine to predict prognosis after liver resection for intrahepatic cholangiocarcinoma. BMC Cancer (2022) 22:258. doi: 10.1186/s12885-022-09352-3 35277130 PMC8915487

[26] van der Ploeg T Austin PC Steyerberg EW. Modern modelling techniques are data hungry: a simulation study for predicting dichotomous endpoints. BMC Med Res Method (2014) 14:137. doi: 10.1186/1471-2288-14-137 PMC4289553 25532820

