# Supplementary material for: Development and validation of a machine learning model for survival risk stratification after esophageal cancer surgery
Source: Front Oncol. 2022 Dec 9;12:1068198. doi: 10.3389/fonc.2022.1068198 (PMC9780661; doi:10.3389/fonc.2022.1068198)
Supplement: Supplementary file 1 [file DataSheet_1.docx]

**tree$extract**

[[1]]

Tune result:

Op. pars: minsplit=23; minbucket=12; cp=0.001; maxdepth=30

auc.test.mean=0.7725272,auc.train.mean=0.8055524,acc.test.mean=0.7552941,acc.train.mean=0.7852941,f1.test.mean=0.8159580,f1.train.mean=0.8435111,logloss.test.mean=0.5459575,logloss.train.mean=0.4854177

[[2]]

Tune result:

Op. pars: minsplit=12; minbucket=1; cp=0.001; maxdepth=4

auc.test.mean=0.7737869,auc.train.mean=0.8091305,acc.test.mean=0.7650616,acc.train.mean=0.7957633,f1.test.mean=0.8386124,f1.train.mean=0.8587292,logloss.test.mean=0.7478243,logloss.train.mean=0.4639764

[[3]]

Tune result:

Op. pars: minsplit=12; minbucket=12; cp=0.001; maxdepth=24

auc.test.mean=0.7449585,auc.train.mean=0.8068432,acc.test.mean=0.7482353,acc.train.mean=0.7952941,f1.test.mean=0.8184537,f1.train.mean=0.8522066,logloss.test.mean=0.5406621,logloss.train.mean=0.4732330

[[4]]

Tune result:

Op. pars: minsplit=12; minbucket=1; cp=0.001; maxdepth=7

auc.test.mean=0.7613371,auc.train.mean=0.8326879,acc.test.mean=0.7647059,acc.train.mean=0.8188235,f1.test.mean=0.8312090,f1.train.mean=0.8696011,logloss.test.mean=0.9881381,logloss.train.mean=0.4430366

**> rf$extract**

[[1]]

Tune result:

Op. pars: ntree=679; mtry=1; nodesize=10

auc.test.mean=0.8183153,auc.train.mean=0.8739645,acc.test.mean=0.7364706,acc.train.mean=0.7811765,f1.test.mean=0.8298071,f1.train.mean=0.8575027,logloss.test.mean=0.6262962,logloss.train.mean=0.5755501

[[2]]

Tune result:

Op. pars: ntree=229; mtry=1; nodesize=6

auc.test.mean=0.8208497,auc.train.mean=0.8693309,acc.test.mean=0.7411765,acc.train.mean=0.7911765,f1.test.mean=0.8216366,f1.train.mean=0.8558086,logloss.test.mean=0.6908410,logloss.train.mean=0.6753623

[[3]]

Tune result:

Op. pars: ntree=100; mtry=1; nodesize=6

auc.test.mean=0.8039200,auc.train.mean=0.8482083,acc.test.mean=0.7466484,acc.train.mean=0.7805003,f1.test.mean=0.8333240,f1.train.mean=0.8539008,logloss.test.mean=1.0046803,logloss.train.mean=0.8189713

[[4]]

Tune result:

Op. pars: ntree=164; mtry=1; nodesize=6

auc.test.mean=0.7921430,auc.train.mean=0.8545028,acc.test.mean=0.7411765,acc.train.mean=0.7947059,f1.test.mean=0.8295094,f1.train.mean=0.8638177,logloss.test.mean=1.0074516,logloss.train.mean=0.8856021

**svm$extract**

[[1]]

Tune result:

Op. pars: C=1.5157165665104; sigma=0.0625

auc.test.mean=0.8061901,auc.train.mean=0.8433749,acc.test.mean=0.7411765,acc.train.mean=0.7870588,f1.test.mean=0.8182725,f1.train.mean=0.8489314,logloss.test.mean=0.5300737,logloss.train.mean=0.4843035

[[2]]

Tune result:

Op. pars: C=0.25; sigma=0.25

auc.test.mean=0.8285371,auc.train.mean=0.8642812,acc.test.mean=0.7862927,acc.train.mean=0.7951889,f1.test.mean=0.8370515,f1.train.mean=0.8452163,logloss.test.mean=0.5144723,logloss.train.mean=0.4470703

[[3]]

Tune result:

Op. pars: C=2; sigma=0.03125

auc.test.mean=0.7739012,auc.train.mean=0.8287102,acc.test.mean=0.7341176,acc.train.mean=0.7452941,f1.test.mean=0.8311972,f1.train.mean=0.8375907,logloss.test.mean=0.5517765,logloss.train.mean=0.5268762

[[4]]

Tune result:

Op. pars: C=1.7411011265...; sigma=0.0625

auc.test.mean=0.8275630,auc.train.mean=0.8573018,acc.test.mean=0.7717647,acc.train.mean=0.7964706,f1.test.mean=0.8411868,f1.train.mean=0.8603333,logloss.test.mean=0.4984590,logloss.train.mean=0.4595782

**gbm$extract**

[[1]]

Tune result:

Op. pars: distribution=bernoulli; n.trees=891; interaction.depth=1; n.minobsinnode=4; shrinkage=0.56

auc.test.mean=0.8267260,auc.train.mean=0.8304656,acc.test.mean=0.7741176,acc.train.mean=0.7888235,f1.test.mean=0.8362486,f1.train.mean=0.8488112,logloss.test.mean=0.4810164,logloss.train.mean=0.4703926

[[2]]

Tune result:

Op. pars: distribution=bernoulli; n.trees=20; interaction.depth=3; n.minobsinnode=7; shrinkage=0.12

auc.test.mean=0.8248722,auc.train.mean=0.8523803,acc.test.mean=0.7741176,acc.train.mean=0.7841176,f1.test.mean=0.8397917,f1.train.mean=0.8496161,logloss.test.mean=0.4872413,logloss.train.mean=0.4572681

[[3]]

Tune result:

Op. pars: distribution=bernoulli; n.trees=20; interaction.depth=7; n.minobsinnode=24; shrinkage=0.23

auc.test.mean=0.8332179,auc.train.mean=0.8562107,acc.test.mean=0.7558687,acc.train.mean=0.7922529,f1.test.mean=0.8256315,f1.train.mean=0.8502985,logloss.test.mean=0.4741813,logloss.train.mean=0.4378849

[[4]]

Tune result:

Op. pars: distribution=bernoulli; n.trees=129; interaction.depth=1; n.minobsinnode=20; shrinkage=0.56

auc.test.mean=0.8354181,auc.train.mean=0.8355472,acc.test.mean=0.7600000,acc.train.mean=0.7758824,f1.test.mean=0.8233473,f1.train.mean=0.8376352,logloss.test.mean=0.4868161,logloss.train.mean=0.4721445

**> xg$extract**

[[1]]

Tune result:

Op. pars: nrounds=244; max_depth=3; lambda=0.557; eta=0.0782; subsample=0.277; min_child_weight=1.27; colsample_bytree=0.39

auc.test.mean=0.8097098,auc.train.mean=0.8444449,acc.test.mean=0.7606840,acc.train.mean=0.7998879,f1.test.mean=0.8365861,f1.train.mean=0.8613348,logloss.test.mean=0.4961573,logloss.train.mean=0.4428533

[[2]]

Tune result:

Op. pars: nrounds=200; max_depth=4; lambda=0.559; eta=0.0772; subsample=0.48; min_child_weight=2.33; colsample_bytree=0.3

auc.test.mean=0.8414336,auc.train.mean=0.8523419,acc.test.mean=0.7764706,acc.train.mean=0.7835294,f1.test.mean=0.8321381,f1.train.mean=0.8383412,logloss.test.mean=0.4832686,logloss.train.mean=0.4641555

[[3]]

Tune result:

Op. pars: nrounds=212; max_depth=4; lambda=0.576; eta=0.018; subsample=0.447; min_child_weight=1.6; colsample_bytree=0.396

auc.test.mean=0.8380557,auc.train.mean=0.8545805,acc.test.mean=0.7647059,acc.train.mean=0.7758824,f1.test.mean=0.8290646,f1.train.mean=0.8404828,logloss.test.mean=0.4892076,logloss.train.mean=0.4666739

[[4]]

Tune result:

Op. pars: nrounds=234; max_depth=5; lambda=0.554; eta=0.0686; subsample=0.312; min_child_weight=1.25; colsample_bytree=0.473

auc.test.mean=0.8356263,auc.train.mean=0.8638126,acc.test.mean=0.7788235,acc.train.mean=0.8200000,f1.test.mean=0.8436769,f1.train.mean=0.8752577,logloss.test.mean=0.4750667,logloss.train.mean=0.4160200
